# Mechanical and Electrical Properties of Sulfur-Containing Polymeric Materials Prepared via Inverse Vulcanization †

**DOI:** 10.3390/polym9020059

**Published:** 2017-02-15

**Authors:** Sergej Diez, Alexander Hoefling, Patrick Theato, Werner Pauer

**Affiliations:** Technical and Macromolecular Chemistry, University of Hamburg, Hamburg D-20146, Germany; diez@chemie.uni-hamburg.de (S.D.); alexander.hoefling@chemie.uni-hamburg.de (A.H.); theato@chemie.uni-hamburg.de (P.T.)

**Keywords:** sulfur, inverse vulcanization, polymeric materials, crosslinking, divinylbenzene, 1,3-diisopropenylbenzene, mechanical properties, electrical properties, bulk polymerization

## Abstract

Recently, new methods have been developed for the utilization of elemental sulfur as a feedstock for novel polymeric materials. One promising method is the inverse vulcanization, which is used to prepare polymeric structures derived from sulfur and divinyl comonomers. However, the mechanical and electrical properties of the products are virtually unexplored. Hence, in the present study, we synthesized a 200 g scale of amorphous, hydrophobic as well as translucent, hyperbranched polymeric sulfur networks that provide a high thermal resistance (>220 °C). The polymeric material properties of these sulfur copolymers can be controlled significantly by varying the monomers as well as the feed content. The investigated comonomers are divinylbenzene (DVB) and 1,3-diisopropenylbenzene (DIB). Plastomers with low elastic content and high shape retention containing 12.5%–30% DVB as well as low viscose waxy plastomers with a high flow behavior containing a high DVB content of 30%–35% were obtained. Copolymers with 15%–30% DIB act, on the one hand, as thermoplastics and, on the other hand, as vitreous thermosets with a DIB of 30%–35%. Results of the thermogravimetric analysis (TGA), the dynamic scanning calorimetry (DSC) and mechanical characterization, such as stress–strain experiments and dynamic mechanical thermal analysis, are discussed with the outcome that they support the assumption of a polymeric cross-linked network structure in the form of hyper-branched polymers.

## 1. Introduction

**Figure 1:**
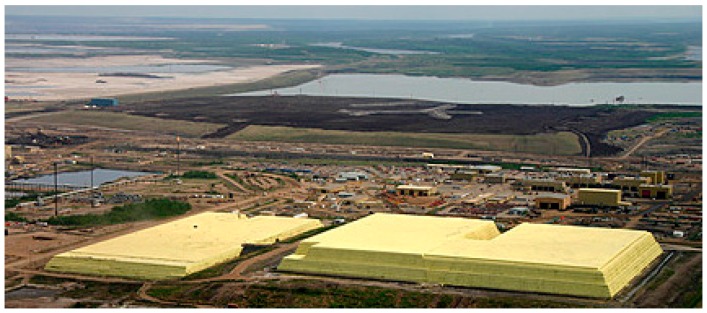
Oil sand sulfur stacks by Syncrude, Athabasca, AB, Canada.

Elemental sulfur is produced on a million ton scale. It is mainly generated as waste by hydrodesulfurization of petroleum in crude oil refineries (>90% recovered in the whole, Reston, VA, USA) [1]. Despite many applications, sulfur world production is projected to reach a surplus of around 70,000 million tons annually, leading to overground storage of sulfur mountains (Figure 1) with unpredictable environmental risks [1]. Its easy availability, low cost and limited applications make sulfur an attractive feedstock for novel materials with a high sulfur content. Currently, elemental sulfur is mainly used for the production of sulfuric acid, which is the resource for many different fine chemical products (battery acid, phosphate fertilizers, ammonium sulfate, disintegrating agents, surfactants for the detergent industry, dyes, etc.) [2].

Sulfur occurs naturally in its thermodynamically most stable modification: orthorhombic sulfur Sα which forms crown-shaped S_8_ rings. Small amounts of S_7_-rings and tiny amounts of other rings also occur. Overall, there are 14 well-known sulfur allotropes whose formation is temperature-dependent [3]. The allotrope polymeric sulfur occupies a special position with its diradical chain-character. The formation takes place by a temperature-initiated ring-opening reaction with subsequent addition of diradical chains (polymerization) [4]. Polymeric sulfur is formed above its floor temperature of 159 °C [5]. Upon further heating, sulfur readily converts to a metastable, one-dimensional elastomer. When exceeding 200 °C, the amount of polymeric species increases but the chain length is reduced [3]. After cooling down to room temperature, the polymeric sulfur and other rings (cyclo-S*_n_*) degrade back to the cyclic S_8_ form (depolymerization) [6,7].

Recently, it was described that sulfur serves as an inexpensive and promising new feedstock for the synthesis of novel polymeric materials. Upon copolymerization with vinyl group containing monomers, the polymeric sulfur is stabilized via a cross-linked structure. Polymeric materials formed by so-called “inverse vulcanization” possess magnificent thermomechanical and electrochemical properties [8]. Because of their high sulfur content, the sulfur comonomers are used as electroactive cathode materials in Li-S batteries (1005 mA∙h∙g^−1^ at 100 cycles [9]). Suitable comonomers for the Li-S battery technology are 1,3-diisopropenylbenzene [8,9,10], divinylbenzene [11,12], styrene (STY) [13], 1,4-diphenylbutadiyne [14], etc. Oleylamine-based sulfur copolymers have been investigated regarding their usage as chalcogenide-semiconductor nanocrystals in the area of nanomaterial synthesis [15,16]. In addition, the use of the nanoparticles (NP) PbS and Au has been extended by Bear et al. to InP/ZnS quantum dots, Fe_3_O_4_ and CoO. The sulfur copolymer with 1,3-diisopropenylbenzene (pS-DIB) is used as a matrix for the inorganic NPs. The resulting sulfur copolymer NP composites show tuneable physical and optical properties, making them particularly suitable for application as optical filters [17]. The incorporation of sulfur moieties into polymers is performed to prepare organic films with high refractive indices for use in optical and optoelectronic technologies [18,19]. The goal is the attainment of high refractive indices for use as waveguiding materials for future optical fiber communication [19]. Applications in concrete production or spraying operations are also conceivable as well as their use in civil engineering regarding the extension to asphalt in road pavements or as insulating material [20]. In this case, sulfur-containing materials are very advantageous due to their resistance towards aqueous acids and concentrated salt solutions [20]. Recently, high porous polymers have been generated from high-sulfur inverse vulcanized copolymers by supercritical CO_2_ compression (scCO_2_). These foams (sulfur copolymers based on DIB, limonene, etc.) have the potential to act as adsorbent material in the area of gas storage and separation [21] and to absorb toxic pollutants from drinking water (mercury capture) [22,23].

Among the first vinyl monomers used for copolymerization with elemental sulfur were dicyciopentadiene and styrene. Blight investigated the structural elucidation [24]. The reaction was carried out at a temperature of 140 °C with varying reaction times. Kim et al. worked out a general synthetic strategy to prepare polymer networks of poly(OLA-*r*-S) copolymers containing PbS nanoparticles [15]. These well-defined PbS/poly(OLA-*r*-S) nanocomposites were synthesized in a one-pot synthesis containing sulfur, oleylamine and PbCl_2_ in 1,2-dichlorobenzene. In addition, free radical copolymerizations of cyclic aryl disulfides and elemental sulfur were carried out in solution and in bulk polymerization [25]. This reaction represents a ring-opening polymerization of cyclic disulfide oligomers prepared by oxidative coupling of aromatic dithiols which form high molecular and linear poly(arylene sulfane)s with a high sulfur content. Equally, sulfur polymers can be synthesized by anionic copolymerization of cyclic sulfides at high temperatures (159 °C) [26,27]. In addition, with the aim of extending the green chemistry to the cross-linker, Parker et al. described the use of low cost and renewable cross-linking monomers with several unsaturated bonds, such as limonene, farnesol, farnesene and myrcene. These alternative cross-linkers have a comparatively lower molecular weight and lower *T*_g_ concerning conventional cross-linkers (DIB, DVB, dicylopentadiene) [23].

By varying the vinyl monomers and the sulfur content, the physical and chemical properties of the produced polymer can be tuned. The target is the synthesis of processable polymers with high chemical stability and with good mechanical and electrical properties. From this perspective, it would be economically and ecologically sustainable to use the excess of elemental sulfur of the hydrodesulfurization for the synthesis of advanced materials.

## 2. Materials and Methods

### 2.1. General Procedure for Bulk Copolymerization in a Pressure Vessel

The reaction was performed in a pressure vessel equipped with a pressure gauge, a safety valve (<10 bar) and a ball valve. During the copolymerization, a constant reaction mass of 200 g in the Polytetrafluoroethylene (PTFE)-inlay was used. Both comonomers (S_8_ and DVB/DIB) were incorporated into the PTFE-inlay with a Komet™ magnetic stir bar (VWR International GmbH, Darmstadt, Germany). The content of used vinyl monomers varied between a content of 10 and 35 wt % in 5 wt % steps. Inverse vulcanization occurred in a closed system without intake of air. The vessel heated to 160 °C and then started the time. The reaction time was 90 min at a stirring speed of 350 rpm. The temperature was approx. 160 ± 5 °C and was controlled by an oil bath (Marlotherm SH, Sasol Germany GmbH, Hamburg, Germany). The product was immediately removed after 90 min reaction.

### 2.2. General Procedure for the Preparation of the Polymers for Analytical Measurements

After the reaction, the sulfur copolymer was crushed and pulverized with an agate mortar to homogenize the product for analytical measurements, such as TGA, DSC and Powder X-ray Diffraction (PXRD). To enable the comminution, liquid nitrogen was used to cool down the polymer to a brittle state. The powder also served as source material for further processing with the hot press.

### 2.3. General Procedure for the Processing of the Sulfur Copolymer with the Hot Press

The processing of the material is performed by means of a hot press. To ensure better removal, PTFE plates and molds with a rectangular cut were used. The powdered sample (about 35 g) of sulfur copolymers was placed in the rectangular form (150 × 150 mm^2^) with a thickness of 2 mm. Afterwards, a temperature of 150 °C and a pressure of 40 ± 10 bar for 20 min was applied to prepare films by compression moulding for different analytical measurements (tensile and electrical tests).

### 2.4. Chemicals and Characterization Methods

Sulfur (colloidal powder, 99.5%, Carl Roth GmbH&Co. KG, Karlsruhe, Germany) and the vinyl monomers 1,3-diisopropenyl benzene (97%, abcr GmbH, Karlsruhe, Germany) and/or divinylbenzene (DVB, 80%, technical grade, Sigma-Aldrich Chemie GmbH, Taufkirchen, Germany) and styrene (freshly distilled, HRC chemicals, Purmerend, The Netherlands) were used as received without further purification.

Gas Chromatography (GC) measurements were conducted by a Agilent 7820A gas chromatograph (Sim GmbH, Oberhausen, Germany) using tetrahydrofuran (HPLC grade) as a solvent and toluene as internal standard with the column CP7778 (50 m length, CP Wax 58 FFAP, Agilent, J&W GC Columns, Frankfurt, Germany) at a column pressure of 50 kPa with a flame ionization detector (FID). Hydrogen was used as mobile phase. 

Differential Scanning Calorimetry (DSC) was performed on a DSC 1 thermal analysis system (Mettler-Toledo GmbH, Gießen, Germany) at a heating and cooling rate of 10 °C∙min^−1^ over the range of −50 to 150 °C under nitrogen atmosphere and the mid-points of the transitions were taken as *T*_g_.

Thermal Gravimetric Analysis (TGA) was carried out by a TGA 7 (PerkinElmer LAS GmbH, Rodgau, Germany) at a heating rate of 10 °C∙min^−1^ under nitrogen atmosphere up to 800 °C. 

Elemental Analysis (Euro EA) was performed using an inductively coupled plasma optical emission spectrometry (ICP-OES) (HEKAtech GmbH, Wegberg, Germany) with Carbon (C), Hydrogen (H), Nitrogen (N) and Sulfur (S), CHNS–Porapack PQS columns.

PXRD measurements were obtained using an X’Pert Pro MPD model (PANalytical GmbH, Kassel, Germany) at room temperature with a Cu-K_α_ radiation source at 40 mA.

Scanning electron microscopy (SEM) images were recorded on a Field Emission Scanning Electron Microscope (LEO 1525 FEG SEM, Carl Zeiss Microscopy GmbH, Jena, Germany) with an In-lens detector. An EHT of 5 kV and a WD of 5 mm was used. The samples for SEM were attached on a carbon surface with a carbon adhesive and vaporized with carbon. 

Tensile Experiments were performed on a materials testing machine from Zwick (Zwick GmbH&Co., Ulm, Germany) at a temperature of 25 °C. Samples were clamped tightly between the two jaws. A camera was fixed in front of the two jaws, to record the elongation process. The tensile test was carried out according to the international norm DIN EN ISO 527-2 with a multipurpose test specimen 5A.

Dynamic Mechanical Analysis (DMA) was carried out on a HAAKE CTC from Thermo Fisher Scientific (Thermo Fisher Scientific GmbH, Karlsruhe, Germany). The storage moduli (*E*’), loss moduli (*E*”), and loss tangents (tan δ) were obtained in the dual cantilever bending mode as a function of temperature over a range of −50–150 °C. The heating rate was 2 °C∙min^−1^ and the frequencies were 0.3, 1, and 10 Hz. The test was performed according to DIN EN ISO 6721 with a multipurpose test specimen 5A without grip section.

The specific contact resistance (Ω∙cm) and the dielectric strength (kV∙mm^−1^) were measured by the company ELANTAS Europe GmbH (Hamburg, Germany). For the electrical measurements, blanks with a size of 10 × 10 mm^2^ are required for the first method and blanks with a size of 40 × 40 mm^2^ for the second method.

## 3. Results and Discussion

This work reports on the properties of sulfur copolymers with different divinyl monomers as cross-linker. The vinyl monomers are based on an aryl scaffolding. The general synthetic approach is to use bulk polymerization without any solvent or additive. The temperature is set to the floor temperature of sulfur (159 °C), because too high temperature would promote the autoacceleration. The time is set to 90 min at a scale of 200 g; this setting is also not chosen too high because it could lead to an expediting of the depolymerization process [10]. In contrast to Pyun, the polymerization occurs in a hermetically sealed system with PTFE inlay (see the Experimental section).This simple process yields colored (yellow-amber and red) hydrophobic and translucent materials with different ductilities or malleabilities.

Depending on the comonomer type or feed, hyper-branched sulfur-containing polymers are obtained which are predominantly thermoplastics with more or less good shape retention. There are, however, influencing factors, such as the gel effect (chain reduction), yielding highly viscous plastomers of the sulfur copolymer with divinylbenzene (pS-DVB), or the impact of intensive heat treatment by the hot press (post cross-linking) leading to brittle thermosettings (pS-DIB). Only high comonomer contents of 30%–35% are affected by this. pS-DIB can form at high temperatures above 100 °C, a highly viscous melt that enables self-healing properties, while pS-DVB exhibits a permanent network, and it can only soften on the surface and is more difficult to process.

### 3.1. Processing

**Figure 2:**
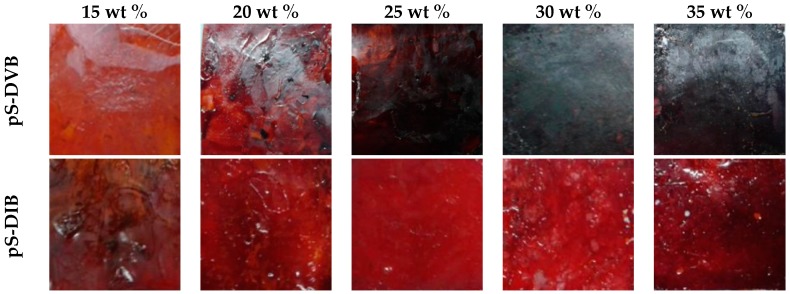
Illustrations of pS-DVB- (**above**) and pS-DIB-films (**below**) obtained by hot press treatment with varying incorporated monomer content.

The processing of hyper-branched copolymers is challenging and requires more than just heat. The copolymer lumps can be milled into a fine powder using an agate mortar and afterwards pressed into films by a hot press. Upon subsequent processing with heat and pressure of the hyper-branched copolymer, glassy thermosets are formed at pS-DIB with higher incorporated DIB (≥30 wt %) by post cross-linking. This process strengthens the material. However, sulfur copolymers with a higher content of incorporated vinyl monomers facilitate the formation of the materials into thermosets upon thermal treatment. The produced films (Figure 2) can be used for further analytical methods.

### 3.2. Structure and Surface

Sulfur copolymers are completely amorphous materials. Nevertheless, the polymer networks can show slight crystalline reflections in the PXRD. The detected attenuated crystalline reflections are in agreement with the powder X-ray diffractogram of crystalline elemental sulfur. In the case of low residual elemental sulfur in all sulfur copolymers, the respective monomers have not been converted completely and the depolymerization occurs with a temporal dependence. The small quantities of sulfur have no influence on thermal or mechanical properties. S_8_ can be qualitatively detected in the first heating phase of the DSC measurement and, in very small amounts, in the SEM images and PXRD diffractograms.

**Figure 3:**
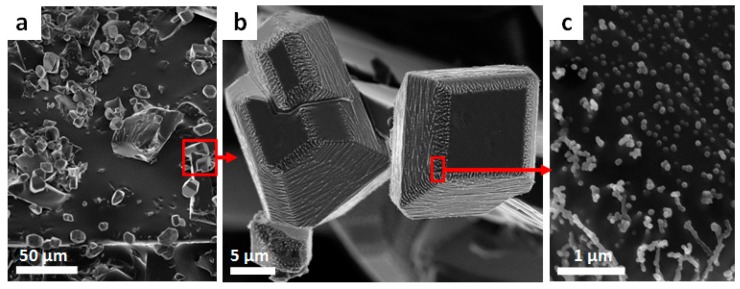
SEM-image of pS-DVB with 30 wt % DVB: (**a**) section with sulfur crystals and agglomerates; (**b**) sulfur crystals with spherical dots; (**c**) spherical dots on sulfur crystals and copolymer surface.

SEM-images of sulfur copolymers show sulfur microcrystals (Figure 3b). These are characterized by an angular shape (Figure 3b) with a plain surface showing little spherical dots (Figure 3c). The microcrystals form agglomerates (Figure 3a). The image shows exemplarily a section with an S_8_ that remains on the polymer and is not distributed over the entire surface of the copolymers. This observation is confirmed by Salman et al. [28].

**Figure 4:**
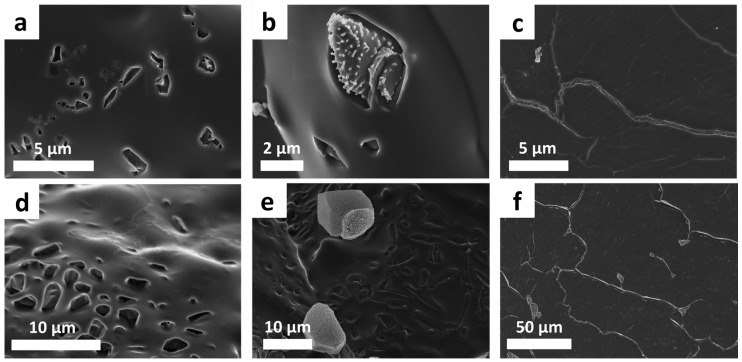
SEM-image of a crude sulfur copolymer with holes and plain surface (**a**,**d**); crude sulfur copolymer with sulfur crystals (**b,e**) and heated copolymer with cracks; treated with the hot press (**c**,**f**). Above pS-DVB is shown, and below pS-DIB is shown with 15 wt % vinyl monomer.

The sulfur copolymer films exhibit a plain surface, partly with unevenly distributed holes to rod-shaped notches (Figure 4a,d). The holes are probably the remainders of sulfur-occupied gaps (Figure 4b,e). After usage of the hot press, elemental sulfur almost disappears, probably by post cross-linking (virtually full conversion of sulfur). The sulfur copolymers show thin cracks when using the hot press (Figure 4c,f).

Consequently, the structure appears slightly inhomogeneous due to residual elemental sulfur, holes, pores and cracks. The linking agents DVB and DIB seem to be incorporated completely into the polymer network because no residual monomer is detected in the gas chromatography. However, low double bonds are available (solid-state NMR), which show the presence of a few non-converted double bonds, so probably only one functional group of the divinyl monomers is affected. This observation of incomplete conversion of double bonds of the vinyl monomers agrees with the investigations of Pyun using larger reaction scales [10]. Despite the same conditions for each reaction and thus similar conversion values of double bonds, other effects (gel effect, post cross-linking) have an additional influence on the properties, possibly with no linear correlation.

### 3.3. Thermal Properties

**Figure 5:**
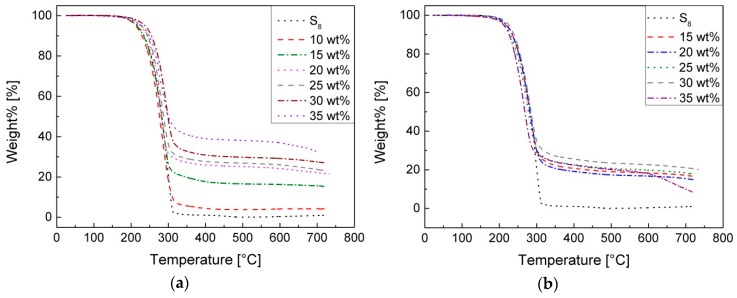
TGA-thermograms with varying incorporated monomer content: (**a**) pS-DVB; (**b**) pS-DIB.

The thermogravimetric analysis (Figure 5) shows thermal resistance up to 220 °C of all types of synthesized sulfur copolymers. Between 200–300 °C, there is a steep, massive weight% loss which is related to the decomposition of the sulfur. By increasing the temperature, no further mass loss occurs. The residues of the pS-DVBs correlate with the respective incorporated monomer content. The residue of pS-DIBs seems to be independent of the incorporated monomer content. The mass residue has a constant value of 17% ± 2%. The high residue after the decomposition indicates that presumably a cross-linking network is present. 

In comparison with the results of Pyun, the decomposition curve of pS-DVB is shifted to a higher temperature (+20 °C), which indicates a higher temperature stability [11]. The course of the decomposition curve of the pS-DIB is neither comparable nor the uniform residue explainable without any dependence of the monomer content. The decomposition temperature corresponds to 5% weight loss of the initial mass, when the decomposition starts. This value occurred in all samples at an average temperature of 222 ± 9 °C (Table 1). Different publications report on similar results above 200 °C of the decomposition temperature [8,11,14].

Bear et al. have analyzed the leaching out in the annealing process between 300–400 °C by means of EDS (Energy-dispersive X-ray spectroscopy) and have attributed it to the decrease of sulfur. Independent of the DIB content, the decay contains approximately 90 wt % sulfur. By means of the supported quantitative XPS analysis (X-ray photoelectron spectroscopy), it was determined that the remaining sulfur does not alter significantly after annealing. In the remaining compound, organic sulfate groups (168.0 eV) were identified, including such species such as thioethers or disulfides (164.0 eV), C–S (163.98 eV), SO_x_ and R–SO_x_–R′ (167.88 eV) as well as C=S (161.5 eV, low intensity). With 80% of the sulfur signals, the C–S bonds have the highest quantity, even though they represent only an average of 8% of the total remaining structure. This indicates an “inverse vulcanization” architecture of the copolymer by retention after the annealing process. Independent of the sample, there is a critical amount of sulfur between 7.4%–14.1%, which remains after annealing. However, the carbon composition fluctuates very strongly. Probably, more stable mono- or disulfide linkages are formed, which stabilize the remaining sulfur content, and a higher initial sulfur concentration preferentially forms longer polysulfide chains (S–S_n_–S) between the –C–S linkages. The longer chains containing S–S bonds tend to decompose at high temperatures under sublimation.

**Figure 6:**
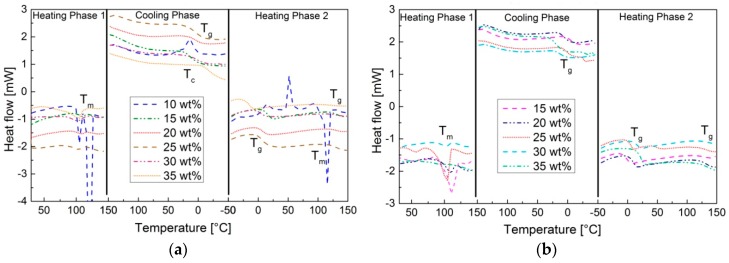
DSC-thermogram: (**a**) pS-DVB_10–35_; (**b**) pS-DIB_15–35_. Representation of both heating steps and of the cooling step. Heating and cooling rate: 10 °C∙min^−1^.

The observed melting point in the first heating step in the DSC-thermogram of pS-DVB (<15 wt % DVB, Figure 6a) and pS-DIB (15–25 wt % DIB, Figure 6b) is caused by small amounts of non- converted elemental sulfur. This residue does not crystallize in the cooling step, so neither *T*_c_ nor *T*_m_ appear afterwards, which shows that a complete S_8_-conversion has taken place after the thermal treatment. *T*_g_ and other transitions are determined for all sulfur copolymers in the second heating step (Figure 6).

**Figure 7:**
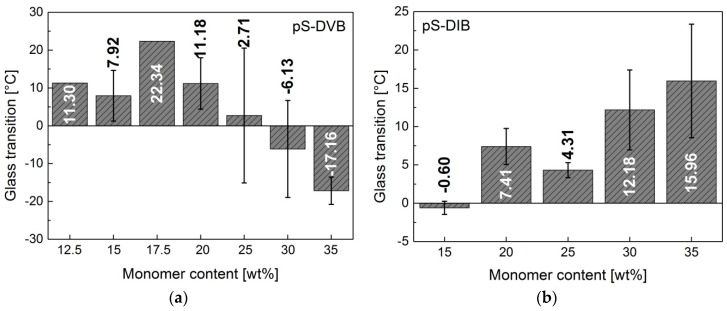
Glass transition *T*_g_ of the sulfur copolymers. (**a**) pS-DVB; (**b**) pS-DIB.

The more pronounced first step, the *T*_g_, shows the transition from glass to plastic in the pS-DVB and from brittle to thermoplastic in the pS-DIB. The *T*_g_ increases by higher cross-linking density or molecular weight as shown in pS-DIB (Figure 7b). pS-DVB (Figure 7a) does not behave accordingly. From an amount of 17.5 wt % DVB, the *T*_g_ tends to decrease with an increase of added DVB content. At a lower scale, an increase in *T*_g_ with increasing monomer content [11] can be observed in the pS-DVB. This divergence, which occurs in comparison to a 200 g scale, can be explained by the chain reduction of pS-DVB (≥30 wt % DVB) caused by exceeding a temperature of 200 °C (gel effect) [3]. Likewise, in the case of a higher DIB content, the *T*_g_ is higher at a lower scale [8,10].

**Figure 8:**
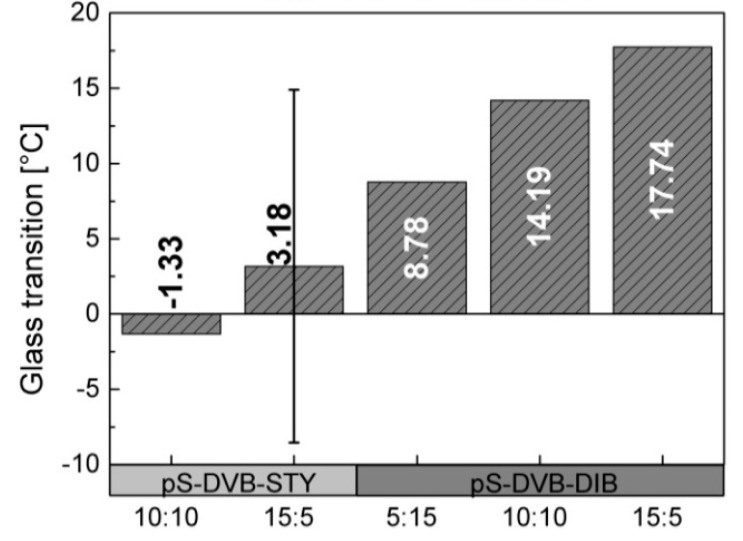
Glass transition of terpolymer based on pS-DVB with STY and DIB. The monomer content amounts to 20 wt %.

The *T*_g_ of the terpolymer tends to increase when the added styrene content is decreased. The decrease of the DIB content in the DVB-based terpolymers leads to an increase of *T*_g_ due to higher cross-linking abilities of DVB in comparison with DIB monomers (Figure 8). Consequently, a higher cross-linking density leads to a higher *T*_g_ [29]. The work of Parker confirmed that styrene reduces the *T*_g_ (similar to DIB) due to its relatively low *T*_g_ and viscous, flexible ductility. Consequently, it shows properties of a plasticizer [13].

The second lower transition could not be assigned, but its presence can be confirmed by the thermogram in the Dynamic Mechanical Analysis (DMA). This deformation transition occurs in pS-DVB as a softening point and in pS-DIB as a transition to a viscous melt. This occurs for all polymers at a temperature of approx. 131.5 ± 1 °C.

To become a viscous melt, despite the cross-linked structure, allows pS-DIB to act as a self-healing material. This behavior occurs due to the lower dissociation energy of S–S bonds (dynamic covalent bonds) in longer S−S chains (33 kcal∙mol^−1^) [30]. Upon thermal activation, the microstructure of the sulfur copolymer backbone cleaves and reorganizes itself into a different macromolecular framework. pS-DVBs exhibit only a slight softening point at high temperatures (>130 °C) without a molten state. Thus, pS-DVBs provide excellent properties in terms of temperature resistance and shape retention. The properties range from thermosetting regarding the cross-linking to plastic regarding the ductility.

### 3.4. Electrical Properties

**Figure 9:**
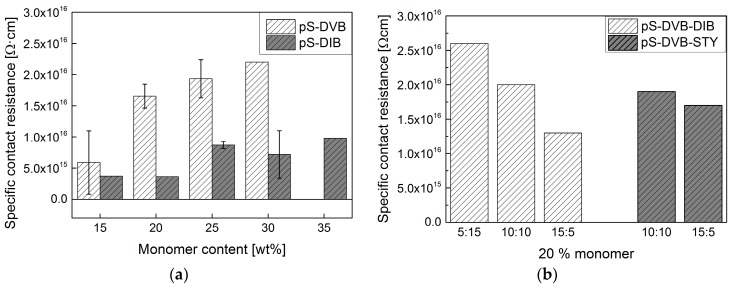
Specific contact resistance of sulfur materials: (**a**) copolymers; (**b**) terpolymers. Measured after 0–3 months of synthesis.

All synthesized types of sulfur copolymers have exceptionally good insulating properties. The specific contact resistance ranges from 10^15^–10^16^ Ω∙cm (Figure 9). Thereby, they provide similarly good properties as previously known insulating materials, such as conventional hydrocarbons like PMMA with 10^15^ Ω∙cm or PE, PP and PB with 10^16^ Ω∙cm [31,32]. The specific contact resistance of sulfur copolymers is one order of magnitude higher than that of elemental sulfur with 10^15^ Ω∙cm. Furthermore, sulfur copolymer materials have a much higher resistivity than the brittle elemental sulfur that does not have any ductility. Thus, sulfur copolymers would be a good alternative for insulating materials with high amounts of inexpensive elemental sulfur. Only materials such as PTFE have a higher specific contact resistance of 10^17^ Ω∙cm [33].

The specific contact resistance of pS-DVB increases with increasing cross-linker DVB (Figure 9a). Thus, a higher DVB content increases the insulating properties. The pS-DIB does not show any correlation with added DIB content and lies in the same order of magnitude (Figure 9a). In this connection, the terpolymer pS-DVB-DIB elucidates the correlation of the increasing specific contact resistance with an increasing DVB content (Figure 9b). In contrast to this, the terpolymer pS-DVB-STY (Figure 9b) refutes the insight that there is an influence on the insulating properties. Certainly, its value is similar and also the influence of the styrene (STY) could be higher than that of DVB.

The resistance of insulating sulfur copolymers against high voltage or dielectric strength is very low. The value of dielectric strength of all test specimens is about 9 kV∙mm^−1^, similar to the value of glass (10 kV∙mm^−1^). Conventional polymers (hydrocarbons) have a dielectric strength of one to two orders of magnitude higher than sulfur copolymers [31]. This test is very sensitive towards air inclusions and inhomogeneity. Whether these low values are due to the material itself or caused by the imperfections of the test specimens could not be resolved definitively. It indicates that the presence of elemental sulfur in the copolymer strongly affects its stability and the reproducibility of the results obtained by different analytical methods. If these negative side effects could be eliminated, the sulfur copolymers with STY and DVB contents in particular would have the potential to be well insulating materials.

### 3.5. Mechanical Properties

**Figure 10:**
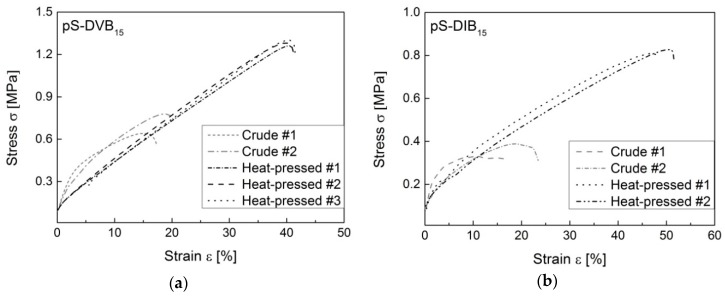
Comparison of ductility experiments of crude and hot-pressed sulfur copolymers. The diagrams show copolymers with 15 wt % incorporated monomer content: (**a**) pS-DVB; (**b**) pS-DIB.

The mechanical tests are very important to characterize the virtually unexplored ductile properties of the products synthesized by inverse vulcanization. Consequently, it is possible to tune these materials for specific applications. All mechanical measurements were done with hot-pressed sulfur copolymers because it is often nearly impossible to reprocess the crude polymers into test specimens. Ductile experiments indicate that hot-pressed sulfur copolymers have better ductile properties than crude copolymers (Figure 10). They show a two to three times higher elongation. Hence, the remolded material has a higher stability for different applications. Attention should be paid to the heating time to prevent the formation of thermosets due to post cross-linking.

**Figure 11:**
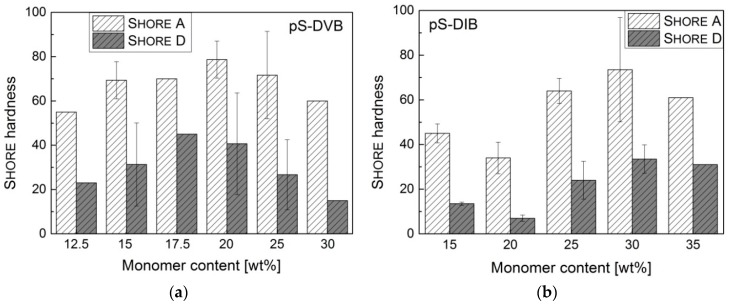
Shore hardness of sulfur copolymers: (**a**) pS-DVB; (**b**) pS-DIB.

The Shore hardness of all sulfur copolymers (reprocessed with a hot press) has no clear correlation with the incorporated monomer content or cannot be detected due to the large standard deviations. The deviations of measurements are caused by inhomogeneities of the sulfur copolymers through air inclusions or the unsuccessful uniform reprocessing with the hot press. The values of the Shore hardness correspond to those of soft plastics or elastomers (Figure 11). Films of pS-DIB are relatively softer than those of pS-DVB (Shore *D* = 13–67) below an incorporated monomer content of 30 wt %. Regarding the Shore hardness, the sulfur copolymer materials are softer than conventional hydrocarbon polymers like polystyrene (*D* = 80), polymethylmethacrylate (PMMA) (*D* = 87–88), polycarbonate (*D* = 82–85), polypropylene (*D* = 65–75), etc. [34].

**Figure 12:**
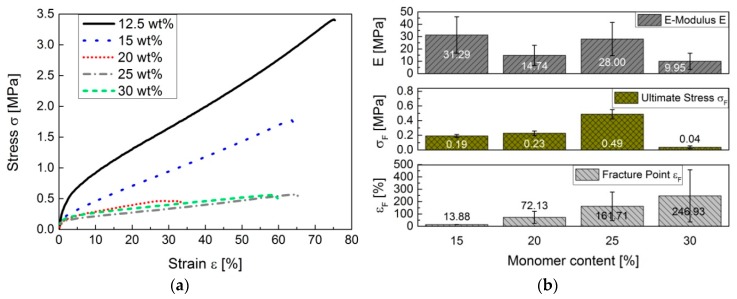
Ductility experiments of the pS-DVB specimens: (**a**) stress–strain diagram; (**b**) ductility parameters (*E*-Modulus *E*, Ultimate Stress *σ_F_* and Fracture Point *ε_F_*).

Depending on the amount of styrene and DIB added to the DVB-based copolymer to form a terpolymer, a softer material is produced in contrast to conventional pS-DVB. Results of ductility experiments show that test specimens of pS-DVB act as ductile material with properties of amorphous plastomers. The curves of ductility experiments (Figure 12a) have a small elastic range and a large plastic range with no necking area. The ductile stress to stretch the polymer by the same factor increases with the decrease of the incorporated monomer content of the sulfur copolymer. This implies that a polymer with a lower incorporated monomer content has a higher stability. At a monomer content of 30–35 wt %, the polymers result in a sticky and viscous resin because a higher monomer content implies an autoacceleration (gel effect) that causes a temperature rise above 200 °C. The released exothermic energy decreases the viscosity by reducing the chain length when the temperature limit is exceeded to a value above 200 °C [3]. These materials cannot be processed into test specimens to carry out mechanical measurements.

The *E*-modulus tends to decrease with an increase of added monomer content. The hardness of the exceptional copolymer with 15 wt % monomer content cannot be explained. Consequently, at a monomer content of 12.5 wt %, the hardness is highest in the plastic material. The stretching area of pS-DVBs extends up to 75%. Apparently, the fracture point decreases first with an increase of the incorporated monomer content, and then increases again at an incorporated monomer content of 25 wt %, when the viscosity of the products decreases (Figure 12b).

**Figure 13:**
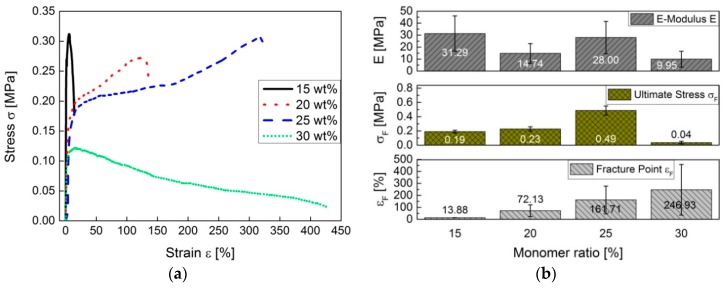
Ductility experiments of the pS-DIB specimens: (**a**) stress–strain diagram; (**b**) ductility parameters (*E*-Modulus *E*, Ultimate Stress *σ_F_* and Fracture Point *ε_F_*).

The material of pS-DIB acts as amorphous plastomers with thermoplastic to duromeric properties, depending on the incorporated monomer content. The stress–strain diagram of pS-DIB shows a plastic region with a necking area (Figure 13a). The material has no elastic properties, since there is no linear elastic range in the stress–strain curve. The elongation increases with an increase of the monomer content added to the sulfur copolymer (Figure 13b). At an incorporated monomer content of 30–35 wt %, most polymers become brittle thermosets (post cross-linking at the hot press), which break under mechanical stress, for instance, during the process of making specimens. The stretching area of pS-DIB extends up to 432%. The fracture point increases continuously with an increase of incorporated monomer content until thermosetting conditions are reached.

Pyun et al. performed tensile tests based on pS-DIB with 20 and 30 wt % monomer content. If their results are compared to the results obtained in this work, the required stress is two orders of magnitude higher and the *E*-modulus is one order of magnitude higher than in the tensile experiments of pS-DIB in this work (Figure 13). These values indicate a material with higher stiffness, and, consequently, a harder material. A monomer content of 20 wt % shows a comparable elongation (100%–250%). However, it is worth mentioning that the elongation of pS-DIB in this work is two orders of magnitude higher for a monomer content of 30 wt % [35] in the event that it is not cross-linked to a thermoset by the processing with the hot press. The curve shape of pS-DIB with 20 wt % corresponds to thermoplastics and the copolymer with 30 wt % DIB corresponds to thermosets [35].

**Figure 14:**
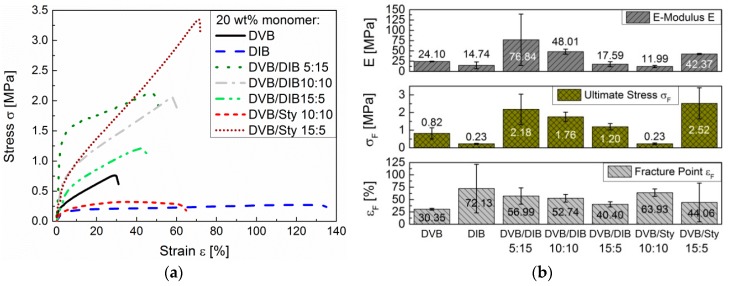
Ductility experiments of terpolymer specimens: (**a**) stress–strain diagram; (**b**) ductility parameters (*E*-Modulus *E*, Ultimate Stress σ*_F_* and Fracture Point ε*_F_*). Constant total incorporated monomer content: 20 wt %.

In comparison, the pS-DIB is more ductile than the pS-DVB, since DIB-based copolymers require 10 times less ductility stress for the same elongation than DVB-based copolymers. An increase of the DIB content in the poly(S-*r*-DVB-*r*-DIB) terpolymer (pS-DVB-DIB) results in an increase of the elongation and of the ultimate stress as well as of the *E*-modulus (Figure 14b). STY acts as a plasticizer (like DIB) and increases the elongation (Figure 14a) when added to the DVB-based terpolymer. The terpolymers contain a total of 20 wt % of incorporated vinyl comonomers with varying ratios.

By the incorporation of a divinylbenzene network, the mechanical strength values are significantly increased and the ductility is decreased. Regarding the mechanical properties, sulfur copolymer materials are softer than conventional polymers. They are characteristic for low toughness and strength and high ductility [31].

**Figure 15:**
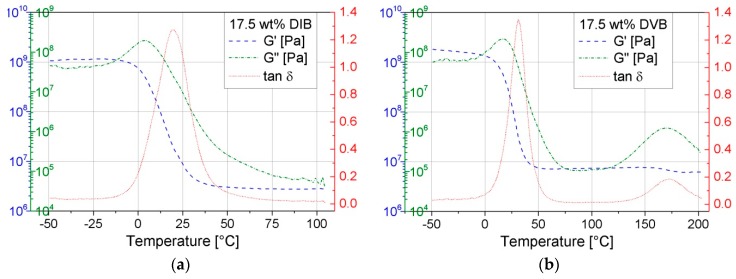
DMA diagrams of sulfur copolymers: (**a**) pS-DIB; (**b**) pS-DVB.

The DMA plots of pS-DVB and pS-DIB (Figure 15) hardly differ in a sulfur copolymer. Only the three respective graphs are shifted in the respective direction (lower or higher temperature), depending on the *T*_g_. Moreover, the terminal plateau of G’ increases with higher incorporated comonomer content, which implies a higher cross-linking degree in the form of hyper-branching.

Above a temperature of approximately −10 °C, the glassy state of the materials ends and the glass transition range begins with a sharp increase of the loss modulus *G*”. Here, the copolymer is increasingly losing its elastic properties and instead a greater conversion of mechanical energy into heat energy takes place. This results in a simultaneous decrease in the storage modulus *G*’. The loss modulus reaches its maximum in the glass transition range. This maximum results from the required energy to increase the chain mobility (molecular friction processes), which is irreversible. 

The maxima of *G*” can be used as reference points to determine relaxations. This applies also to the maximum of tan δ (loss factor). Thus, the first maximum of *G”* is exhibited in the *T*_g_, which corresponds to the results of the DSC. The second weak maximum of *G”* deviates approximately by 30–40 °C from the secondary transition (β-relaxation) in the DSC thermogram. However, the β-relaxation (*G*”) of the pS-DIBs cannot be determined due to impending softening of the material (viscous polymer melt). The results obtained by DSC are in agreement with those of the DMA regarding the *T*_g_. *G*’ is steadily decreasing (sigmoidal decrease) throughout the test period and has a turning point in the glass transition range. The course of *G*’ in the terminal plateau suggests a low altitude, and, consequently, a low cross-linking polymer network, and is confirmed by the results of the stress–strain experiments.

**Figure 16:**
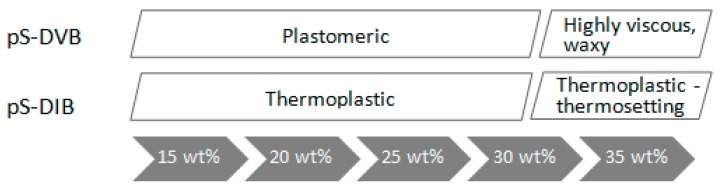
Scheme of the dependence between polymer type and incorporated monomer content.

Finally, the sulfur copolymers can be subdivided into different classes regarding their thermomechanical behavior. The most noticeable behavior can be observed above an incorporated monomer content of 30 wt %, in which case a dramatic change of the mechanical properties of both sulfur materials occurs (Figure 16). In the pS-DVB, the high exothermicity, which is released at a high monomer content (≥30 wt %), is responsible for the property change. In the pS-DIB, however, cross-linking continues upon thermal treatment in the hot press, whereupon a glassy thermosetting occurs.

## 4. Conclusions

This report shows processable sulfur copolymers with exceptional mechanical and insulating properties. The copolymers exhibit different degrees of ductility with an adjustable *T*_g_ between −22–22 °C as well as a specific contact resistance of more than one order of magnitude in comparison with elemental sulfur. The product can be obtained via a straight-forward synthetic route (bulk polymerization) under simple conditions, called inverse vulcanization. Furthermore, all of the products are translucent and hydrophobic. 

A wide range of polymer properties is covered. Poly(S-*r*-DVB) copolymers with 12.5%–30% DVB possess amorphous and plastomeric properties with slight elasticity and a high shape retention. A higher DVB content between 30%–35% results in low viscose, waxy plastomers with a high flow behavior. The properties of the poly(S-*r*-DIB) copolymers range from flexible thermoplastic (15%–30% DIB) to brittle thermosetting (30%–35% DIB). The divergent properties of the respective sulfur copolymers with a high cross-linker content (≥30%) are evoked by effects, such as the gel effect (chain reduction by high exothermicity) in the poly(S-*r*-DVB) copolymer and thermal post-crosslinking (processing at the hot press) in the poly(S-*r*-DIB) copolymer. By addition of a second cross-linker to the comonomers, the ductility can be influenced. A higher DVB content leads to a higher strength and shape retention. The addition of DIB or STY softens up the material or makes it more flexible. The material properties depend largely on the content and on the type of incorporated monomer. 

The processing of the crude sulfur copolymer with the hot press improves the material properties significantly regarding mechanical deformation, and it induces post cross-linking of the remaining elemental sulfur. Therefore, it does not have to be removed separately. After the treatment, the shape retention of the sulfur material was clearly improved.

In all polymers, an incorporated monomer content of 15 wt % yields the best results concerning the visual properties, the ductility and the processing. These novel polymers based on sulfur are potentially very useful materials, resulting in a multitude of new possibilities for the application of sulfur, like thermal or electrical insulation materials for underground cable systems or primary and secondary windings in transformers, optical lenses, seals in civil engineering, etc.

## Figures and Tables

**Table 1:** Decomposition temperature at 5% weight loss (TGA).

Monomer (wt %)	pS-DVB (°C)	pS-DIB (°C)	Terpolymer (monomer content: 20 wt %)		
			Material	*T*_g_ (°C)	Cross-linker ratio
10	212	-	S_8_	216 ± 4	-
15	231 ± 21	226 ± 11	-	216	DVB/DIB 5:15
20	226 ± 8	215 ± 7	pS-DVB-DIB	224	DVB/DIB 10:10
25	227 ± 8	224 ± 14	-	234	DVB/DIB 15:5
30	241 ± 4	221 ± 7	pS-DVB-STY	216	DVB/STY 10:10
35	231 ± 3	212		213 ± 4	DVB/STY 15:5
